# Correction: Pain-related fear of movement dynamics in individuals with and without low back pain participating in weightlifting and/or powerlifting training

**DOI:** 10.1371/journal.pone.0314625

**Published:** 2024-11-22

**Authors:** Bernard X. W. Liew, Josce Syrett, Paul Freeman, David W. Evans

The units in “kg” in Table 2 should have been “%body mass”. Please see the correct Table 2 here.

**Table 2:** Descriptive characteristics of sample.

Variable	N = 67^1^
**Age (yrs)**	27.70 (8.35)
**Gender**	
Female	32 / 67 (48%)
Male	35 / 67 (52%)
**Height (m)**	1.71 (0.09)
**Body mass (kg)**	74.26 (12.22)
**Training sport**	
OWL	20 / 67 (30%)
PL	25 / 67 (37%)
Both	22 / 67 (33%)
**Years of training**	
1–2	39 / 67 (58%)
3–4	28 / 67 (42%)
**Frequency of training (days/week)**	
2	1 / 67 (1.5%)
3	6 / 67 (9.0%)
4	12 / 67 (18%)
5	34 / 67 (51%)
6	9 / 67 (13%)
7	5 / 67 (7.5%)
**Participants with LBP symptoms**	21 (31.3%)
**LBP intensity (0–100)**	12.13 (16.19)
**Back Squat (%body mass)**	170.83 (54.61)
**Deadlift (%body mass)**	201.59 (55.65)
**Bench Press (%body mass)**	104.75 (36.04)

^1^Mean (SD); n / N (%)
