# Supplementary material for: Pain-related fear of movement dynamics in individuals with and without low back pain participating in weightlifting and/or powerlifting training
Source: PLoS One. 2022 Oct 27;17(10):e0276983. doi: 10.1371/journal.pone.0276983 (PMC9612576; doi:10.1371/journal.pone.0276983)
Supplement: S1 Table — (DOCX) [file pone.0276983.s001.docx]

Table S1. Partial correlation values and 95% confidence interval

| node1 | node2 | fitted | q2.5 | q97.5 |
| --- | --- | --- | --- | --- |
| Q14 | Q18 | 0.32 | 0.08 | 0.45 |
| Q3 | Q4 | 0.32 | 0.05 | 0.54 |
| Q2 | Q35 | 0.28 | 0.08 | 0.41 |
| Q4 | Q30 | 0.27 | 0.00 | 0.46 |
| Q23 | Q32 | 0.27 | 0.04 | 0.50 |
| Q8 | Q13 | 0.27 | 0.06 | 0.46 |
| Q13 | Q38 | 0.26 | 0.00 | 0.45 |
| Q8 | Q35 | 0.22 | 0.00 | 0.41 |
| Q2 | Q18 | 0.22 | 0.00 | 0.38 |
| Q3 | Q13 | 0.20 | 0.00 | 0.39 |
| Q14 | Q32 | 0.19 | 0.00 | 0.40 |
| Q18 | Q23 | 0.18 | 0.00 | 0.38 |
| Q30 | Q32 | 0.18 | 0.00 | 0.39 |
| Q14 | Q15 | 0.16 | 0.00 | 0.35 |
| Q2 | Q15 | 0.16 | 0.00 | 0.34 |
| Q23 | Q30 | 0.15 | 0.00 | 0.37 |
| Q2 | Q23 | 0.15 | 0.00 | 0.33 |
| Q8 | Q23 | 0.14 | 0.00 | 0.31 |
| Q35 | Q38 | 0.14 | 0.00 | 0.29 |
| Q15 | Q38 | 0.13 | 0.00 | 0.32 |
| Q3 | Q30 | 0.13 | 0.00 | 0.37 |
| Q8 | Q15 | 0.11 | 0.00 | 0.35 |
| Q4 | Q32 | 0.11 | 0.00 | 0.30 |
| Q14 | Q23 | 0.11 | 0.00 | 0.30 |
| Q13 | Q30 | 0.11 | 0.00 | 0.26 |
| Q8 | Q38 | 0.10 | 0.00 | 0.30 |
| Q18 | Q35 | 0.10 | 0.00 | 0.29 |
| Q14 | Q38 | 0.10 | 0.00 | 0.31 |
| Q13 | Q35 | 0.08 | 0.00 | 0.25 |
| Q8 | Q18 | 0.06 | 0.00 | 0.23 |
| Q2 | Q38 | 0.06 | 0.00 | 0.27 |
| Q15 | Q30 | 0.04 | 0.00 | 0.27 |
| Q4 | Q35 | 0.04 | 0.00 | 0.24 |
| Q4 | Q38 | 0.04 | 0.00 | 0.25 |
| Q30 | Q38 | 0.04 | 0.00 | 0.24 |
| Q2 | Q4 | 0.02 | 0.00 | 0.20 |
| Q18 | Q32 | 0.02 | 0.00 | 0.26 |
| Q8 | Q14 | 0.02 | 0.00 | 0.19 |
| Q2 | Q8 | 0.02 | 0.00 | 0.22 |
| Q4 | Q15 | 0.02 | 0.00 | 0.20 |
| Q3 | Q23 | 0.01 | -0.01 | 0.20 |
| Q13 | Q14 | 0.00 | -0.03 | 0.06 |
| Q13 | Q15 | 0.00 | 0.00 | 0.17 |
| Q13 | Q18 | 0.00 | 0.00 | 0.09 |
| Q13 | Q23 | 0.00 | 0.00 | 0.17 |
| Q13 | Q32 | 0.00 | -0.09 | 0.10 |
| Q14 | Q30 | 0.00 | -0.11 | 0.06 |
| Q14 | Q35 | 0.00 | 0.00 | 0.09 |
| Q15 | Q18 | 0.00 | 0.00 | 0.19 |
| Q15 | Q23 | 0.00 | -0.18 | 0.00 |
| Q15 | Q32 | 0.00 | -0.15 | 0.00 |
| Q15 | Q35 | 0.00 | 0.00 | 0.15 |
| Q18 | Q30 | 0.00 | -0.04 | 0.13 |
| Q18 | Q38 | 0.00 | -0.19 | 0.00 |
| Q2 | Q13 | 0.00 | -0.12 | 0.00 |
| Q2 | Q14 | 0.00 | 0.00 | 0.11 |
| Q2 | Q3 | 0.00 | 0.00 | 0.15 |
| Q2 | Q30 | 0.00 | 0.00 | 0.11 |
| Q2 | Q32 | 0.00 | 0.00 | 0.10 |
| Q23 | Q35 | 0.00 | 0.00 | 0.14 |
| Q23 | Q38 | 0.00 | -0.01 | 0.10 |
| Q3 | Q14 | 0.00 | -0.15 | 0.08 |
| Q3 | Q15 | 0.00 | -0.10 | 0.10 |
| Q3 | Q18 | 0.00 | -0.10 | 0.07 |
| Q3 | Q32 | 0.00 | -0.14 | 0.13 |
| Q3 | Q35 | 0.00 | -0.02 | 0.11 |
| Q3 | Q38 | 0.00 | -0.17 | 0.02 |
| Q3 | Q8 | 0.00 | -0.01 | 0.09 |
| Q30 | Q35 | 0.00 | -0.09 | 0.04 |
| Q32 | Q35 | 0.00 | -0.18 | 0.00 |
| Q32 | Q38 | 0.00 | -0.09 | 0.07 |
| Q4 | Q13 | 0.00 | 0.00 | 0.16 |
| Q4 | Q14 | 0.00 | 0.00 | 0.18 |
| Q4 | Q18 | 0.00 | -0.03 | 0.13 |
| Q4 | Q23 | 0.00 | 0.00 | 0.16 |
| Q4 | Q8 | 0.00 | -0.03 | 0.06 |
| Q8 | Q30 | 0.00 | -0.17 | 0.00 |
| Q8 | Q32 | 0.00 | -0.05 | 0.05 |
